# Visual outcomes endorse surgery of patients with spheno-orbital meningioma with minimal visual impairment or hyperostosis

**DOI:** 10.1007/s00701-020-04554-9

**Published:** 2020-09-04

**Authors:** Amir H. Zamanipoor Najafabadi, Stijn W. Genders, Wouter R. van Furth

**Affiliations:** 3 University Neurosurgical Centre Holland, Leiden University Medical Centre, Haaglanden Medical Centre and Haga Teaching Hospital, Leiden and The Hague, The Netherlands; 1 Department of Ophthalmology, Leiden University Medical Center, Leiden, The Netherlands; 2 Orbital Center, Department of Ophthalmology, Amsterdam University Medical Center, Amsterdam, The Netherlands

**Keywords:** Spheno-orbital, Meningioma, Surgery, Vision, Hyperostosis

## Abstract

**Background:**

Most spheno-orbital meningioma series span multiple decades, and predictors of visual outcomes have not yet been systemically assessed. We describe visual outcomes in a recent cohort and assess predictors of postoperative visual outcomes.

**Methods:**

Consecutive case series operated by a team of a neurosurgeon and orbital surgeon between May 2015 and January 2019. Best corrected visual acuity (BCVA), visual fields (static perimetry), and relative proptosis were measured preoperatively and postoperatively at 3/6/12 months after which it was assessed yearly. Predictors were assessed with linear regression analysis.

**Results:**

Nineteen patients (all WHO grade I) were operated by the pterional approach (median follow-up 2.4 years). Preoperative visual acuity deficits (*n* = 10) normalized in 70% and improved in 10% (median preoperative: 0.8, postoperative: 1.2, *p* = 0.021). Preoperative visual field deficits (*n* = 8) normalized in all patients (preoperative: − 6.5 dB, postoperative: − 1.5 dB, *p* = 0.008). Preoperative proptosis (*n* = 16) normalized in 44% and improved in 56% (preoperative: 5 mm, postoperative: 2 mm, *p* < 0.001). BCVA and visual fields remained stable at longer follow-up in 95% of patients, while 21% showed progression of proptosis. Predictors for worse longer-term (> 12 months) BCVA were worse preoperative BCVA (*p* = 0.002) and diagnosis of multiple meningioma (*p* = 0.021). Predictors for worse longer-term visual fields were higher diameter of hyperostosis (*p* = 0.009) and higher Simpson grade (*p* = 0.032). Predictor for short-term (3 months) proptosis was preoperative proptosis (*p* = 0.006).

**Conclusion:**

We recommend surgery, even of patients with minimal visual impairment or hyperostosis, as patients who present with deteriorated visual function or extensive hyperostosis are less likely to have postoperative visual outcomes restored to normal.

**Electronic supplementary material:**

The online version of this article (10.1007/s00701-020-04554-9) contains supplementary material, which is available to authorized users.

## Introduction

Spheno-orbital meningioma (SOM) are tumors originating from the sphenoid ridge, primarily characterized by hyperostosis of the lesser and/or greater sphenoid wing [22, 28]. In addition, the majority of patients have an intradural meningioma, often described as a thin “carpet-like” or “en-plaque” tumor, which can be more extensive including cavernous sinus involvement and an intraorbital component [21, 23, 28]. Due to its location, the majority of patients present with visual deficits and/or proptosis [30].

Due to the low incidence of SOM, current series in the literature describe smaller and larger patient series often covering multiple decades, while surgical techniques have improved over the years [3, 9, 14–16, 19, 21–23, 28, 31]. In these series, surgery has proven its value with improvement of visual function (10–73%) and proptosis (50–93%) [3, 9, 14–16, 19, 22, 23, 28]. Nevertheless, many papers only describe the preoperative and postoperative visual acuity and proptosis, neglecting patients’ visual fields deficits, while this is strongly associated with patients’ health-related quality of life [9, 15, 16, 19, 21, 23]. In addition, predictors of visual outcomes have not yet been systematically assessed. Identification of these predictors may optimize the decision and timing of surgical treatment and tailor postsurgical ophthalmological follow-up.

Therefore, we aimed to describe visual outcomes, complications and recurrence in a recent cohort of surgically treated SOM patients in a high-volume referral centre with a dedicated multidisciplinary team. In addition, we systematically assessed predictors of short- and longer-term postoperative best corrected visual acuity (BCVA), visual fields, and proptosis.

## Methods

### Study setting and subject selection

Consecutive (i.e., no case selection) spheno-orbital meningioma patients operated between June 2015 and January 2019 in the Leiden University Medical Center (LUMC) in Leiden the Netherlands were described in this study. A set team of a neurosurgeon (WRvF) and orbital/oculoplastic surgeon (SWG) operated patients and followed patients at their multidisciplinary outpatient clinic. SOM was defined as an inner sphenoid-ridge meningioma with hyperostosis of at least the lesser or greater sphenoid wing with an intradurual meningioma. Patients were excluded if previously operated. In our center the usual first line treatment of SOM consists of surgery, with radiotherapy reserved for recurrent tumors. This study was reviewed and approved by the LUMC-LDD medical ethics committee as part of a larger study protocol (G19.011).

### Surgical technique

**Fig. 1:**
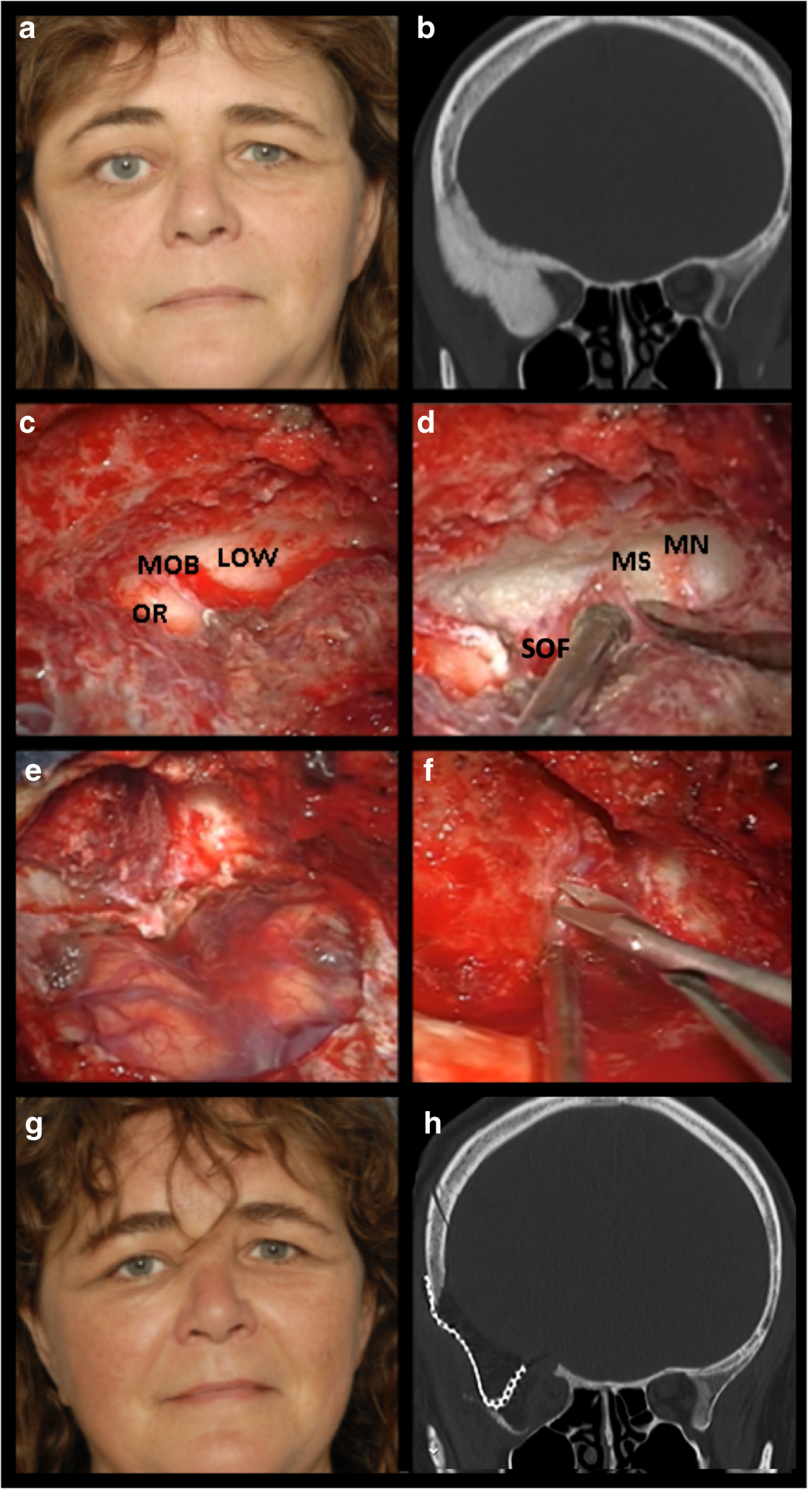
Example of spheno-orbital meningioma patient management. (A) Patient presented with a relative proptosis of 8 mm of the right eye, BCVA of 0.6, and a visual field deficit of − 6.50 dB. (B) Hyperostosis of both the orbital roof and lateral orbital wall is shown on the CT scan in bone setting. (C/D) Pictures of the microsurgical decompression. MOB meningo-orbital band, OR orbital roof, LOW lateral orbital wall, MS maxillary strut, MN maxillary nerve. (E) A Simpson grade I resection was achieved after intradural and intraorbital meningioma resection. (F) After resection of intraorbital meningioma, vertical cuts were made in the periorbita to reduce proposes. (G) Postoperative facial picture showed clear reduction of proptosis. Her BCVA normalized (1.20) as well as the visual field deficit (− 0.33 dB). (H) CT scan in bone setting showed reduction of hyperostotic bone and reconstruction of the lateral orbital wall with titanium mesh. Figures published with permission of the patient after written informed consent

The pterional approach was used in all cases. Patients were positioned in the supine position, with the head extended and rotated to the contralateral side. An interfascial temporal flap was developed to expose the skull [38]. Neuronavigation was used to verify extension of bony resection. Hyperostotic bone of the orbital roof and lateral orbital wall was microscopically decompressed from the maxillary strut to the optic strut using the eggshell technique, which comprises thinning of bone to softly peel the layer of bone around critical structures. If involved the optic canal was decompressed in total length. The meningo-orbital band was cut to fully expose the superior orbital fissure (Fig. 1). Intradural meningioma was removed, but no attempts were made to remove intracavernous sinus meningioma. Intraorbital meningioma was resected by the orbital surgeon, and periorbita was partially resected, or incised, to reduce proptosis. Common grafting techniques (cranial periosteum, donor, or artificial material) were used for watertight dural reconstruction. If indicated, the lateral orbital wall was reconstructed with titanium mesh, or patient-specific 3D-printed PEEK (polyetheretherketone) implant to prevent pulsatile enopthalmos and/or adhesion of the temporal muscle to the periorbita. Abdominal fat or gelatine-based artificial material was used to fill-up the defect. The surgical technique was somewhat modified over time based on developing experiences and new insights.

### Data collection

Demographic characteristics were collected from the electronic patient charts. Patients underwent both computed tomography (CT) and gadolinium contrast-enhanced magnetic resonance imaging (MRI) both before and after surgery (postoperative: after 6 months and then yearly). Multiple visual outcomes were measured preoperatively, and postoperatively at 3, 6, and 12 months, after which patients were seen yearly in the multidisciplinary outpatient clinic of both surgeons. Best corrected visual acuity (BCVA) was measured with the Snellen chart. Patient’s visual fields were investigated using the Zeiss Humphrey visual field analyzer, described as mean deviation (MD) in decibel (dB). Proptosis was determined by measuring axial globe position using a double-prism exopthalmometer, comparing the affected eye with the unaffected eye [11].

### Statistical analysis

Outcomes after surgery are described as the percentage of patients with deteriorated, stable, improved or normalized BCVA, visual fields, and proptosis. Preoperative outcomes were compared with direct postoperative outcomes using the Wilcoxon signed rank test. Individual patient data are graphically depicted over time for all outcomes in graphs. Furthermore, median values were calculated for all patients together and for those patients with and without preoperative visual acuity deficits (cut-off for deficit 0.8 or lower), visual field deficits (cut-off for deficit -5 dB or lower) [26], or proptosis (cut-off for clinically relevant proptosis 2 mm or more). No cut-offs for improvement on the individual patient level were set, as clinical interpretation of improvement is highly dependent on the preoperative status (e.g., visual acuity improvement of 0.0 to 0.4 vs 1.0 to 1.4). Instead, the above-mentioned cut-offs were used both preoperatively and postoperatively and distinction was made between postoperative improvement and normalization of visual outcomes. Predictors of BCVA, visual fields and proptosis were assessed by univariable linear regression analysis, separately for the direct postoperative outcomes (3 months) and outcomes at longest follow-up. No multivariable analysis was performed due to the small number of patients. IBM SPSS Statistics version 23.0 (Armonk, NY, USA) was used for all statistics, and a *p* < 0.05 was considered statistically significant.

## Results

### Subjects

During the study period, 20 patients were operated, but one patient was lost to follow-up, as the patient died due to comorbidities not related to the SOM or surgery. The remaining 19 patients were described in this study (median age: 47.0, 97% female). All patients suffered from unilateral disease. See Table 1 for a description of all baseline characteristics. Median follow-up time between diagnosis and surgery was 7.2 months, as a short wait-and scan regimen was chosen as initial treatment for patients who only presented with proptosis without any visual deficits. Median follow-up time after surgery was 2.4 years (IQR 1.3 to 3.3).

**Table 1:** Baseline characteristics of Spheno-orbital meningioma patients

	LUMC cohort (*n* = 19)
Gender, female	18 (95%)
Age at surgery, years	47.0 (45.0–50.0)
Time between diagnosis and surgery in months	7.2 (3.4–8.9)
Hyperostosis diameter (mm)	31.0 (24.0–35.0)
Soft tissue diameter (mm)	11.0 (8.0–18.0)
Simpson grade	
Grade I	6 (32%)
Grade II	9 (47%)
Grade III	0 (0%)
Grade IV	4 (21%)
Extent of resection	
Full resection	15 (79%)
Subtotal resection	4 (21%)
WHO grade I	19 (100%)
WHO subtypes	
Meningothelial	15 (79%)
Transitional	3 (16%)
Secretory	1 (5%)
Number of tumors	
1	13 (69%)
2	3 (16%)
3	0 (0%)
4	1 (5%)
5	2 (11%)
Postoperative proton radiotherapy	2 (11%)
Postoperative photon radiotherapy	1 (5%)
Reoperation	2 (11%)
Follow-up length in years	2.4 (1.3–3.3)

Continuous outcomes are described as median value and interquartile range. Dichotomous outcomes are described as number and percentages. Percentages might not add up to 100% due to rounding

Extent of resection was determined intraoperatively and on postoperative CT and MRI scan. A subtotal resection was defined as residual meningioma tissue or hyperostosis

### Surgical techniques

In all cases the pterional approach was used, including decompression of the lateral orbital wall and superiorior orbital fissure (Table 2). The principles of the used surgical technique modified somewhat over time; the meningo-orbital band was cut in the last 10 patients (38%) to facilitate full exposure of the superior orbital fissure. Furthermore, in the first couple of operated patients the optic canal and orbital roof were only decompressed if preoperative CT showed extensive hyperostosis of these structures and/or a patient presented with visual acuity or visual field deficits. In the last 12 patients the orbital roof and optic canal were decompressed in all patients. Resection of the anterior clinoid process and decompression of the foramen rotundum, ovale, and spinosum were only performed when clinically indicated. In the first patients, reconstruction of the lateral orbital wall was performed with titanium mesh, while in recent patients patient-specific 3D-printed PEEK implants were used for reconstruction. Gross total resection, i.e., resection of meningioma tissue and hyperostotic bone, was achieved in 14 patients (74%). A subtotal resection was achieved in 5 (26%) patients, due to extensive hyperostosis over the skull base.

**Table 2:** Surgical techniques

	LUMC cohort (*n* = 19)
Resection hyperostotic bone	
Lateral orbital wall	19 (100%)
Orbital roof	
Complete	10 (53%)
Partial	5 (26%)
Not	4 (21%)
Anterior clinoid process	1 (5%)
Decompression of foramina	
Superior orbital fissure	19 (100%)
Optic canal	
Complete (full-length)	7 (37%)
Partial	5 (26%)
Not	7 (37%)
Foramen rotundum	1 (5%)
Foramen ovale	0 (0%)
Foramen spinosum	1 (5%)
Resection of soft-tissue structures	
Meningo-orbital band	10 (53%)
Intraorbital meningioma	10 (53%)
Periorbita management	
Cuts	4 (22%)
Stripping	7 (37%)
Nothing	8 (42%)
Reconstruction	
Patient-specific 3D PEEK implant	3 (16%)
Titanium mesh reconstruction	12 (63%)
No reconstruction performed	4 (21%)
Periumbilical fat filling	11 (58%)

Percentages might not add up to 100% due to rounding

*PEEK* polyetheretherketone

### Visual outcomes

**Fig. 2:**
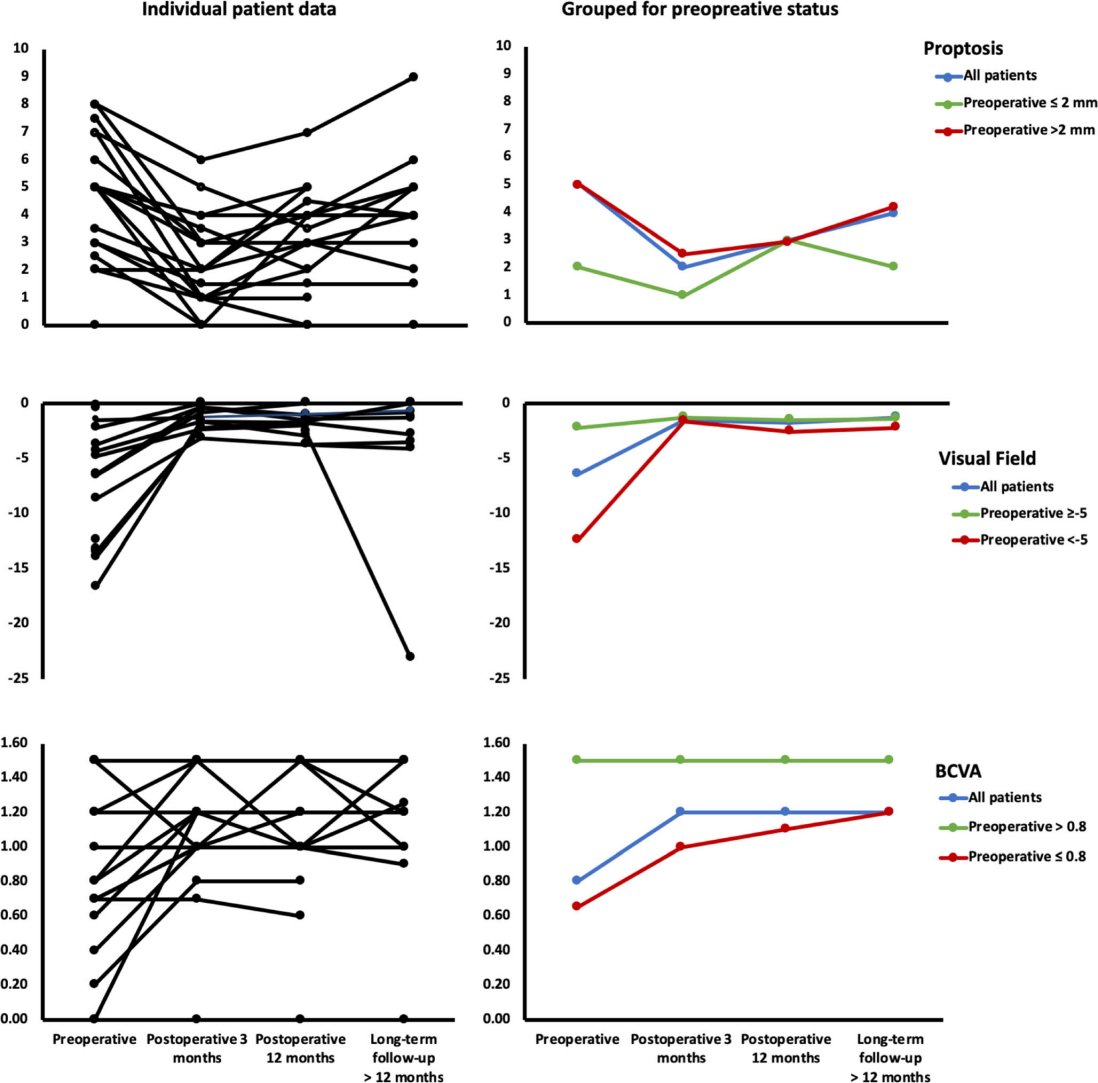
Preoperative and postoperative measures of proptosis, visual fields, and visual acuity are depicted for individual patients and grouped for all patients and patients with and without preoperative deficits. Proptosis was measured with a Hertel exopthalmometer in mm**.** Visual fields were measured with the Humphrey visual field analyzer, described as mean deviation (MD) in decibel (dB). Visual acuity was measured with the Snellen chart

Ten (53%) patients suffered from a decrease in BCVA, which normalized in 7 (70%) after surgery, improved in 1 (10%), and remained unchanged in 2 (20%, preoperative BCVA: 0.0 and 0.7) patients. Median BCVA before surgery was 0.8 (IQR 0.7 to 1.5), which improved postoperatively to 1.2 (IQR 1.0 to 1.5, *p* = 0.021), and remained stable in all patients at 1-year follow-up (1.2, IQR 1.0 to 1.5) and longer follow-up (1.2, IQR 1.0 to 1.5). Eight (42%) patients had preoperative visual field deficits, which normalized in all (100%) patients after surgery. Median visual field before surgery was − 6.5 dB (IQR − 12.9 to − 3.0), which improved postoperatively to − 1.5 dB (IQR − 2.2 to − 0.7, *p* = 0.03) and remained stable in seven (88%) patients at 1-year follow-up (all patients − 1.7 dB, IQR − 2.5 to − 1.1) and longer follow-up (all patients − 1.3 dB, IQR − 3.2 to − 0.3). One patient suffered from a strong deterioration (− 23.1 dB) after 1-year follow-up. Sixteen (84%) patients presented with proptosis preoperatively, which normalized in seven (44%) and improved in nine (54%) patients. Median relative proptosis before surgery was 5 mm (IQR: 3.0 to 6.5), which improved postoperatively to 2 mm (IQR: 1.0 to 3.3, *p* < 0.01). However, four of these patients (25%) suffered from deterioration at 1-year follow-up (all patients 3 mm, IQR 2 to 4) and one patient (6%) at longer follow-up (all patients 4 mm, IQR 2 to 5). Individual patient data over time of BCVA, visual fields, and proptosis are depicted in Fig. 2. In addition, median values are provided for all patients together and separately for patients with and without preoperative visual acuity deficits, visual field deficits, and proptosis.

### Complications and reintervention

Patients suffered from the following postoperative complications: transient (*n* = 3) and permanent (*n* = 3) hypesthesia of the maxillary nerve, transient deficit of the frontal branch of the facial nerve with consequently asymmetry of the eyebrows (*n* = 3), wound abscess requiring debridement of the wound (*n* = 1), preseptal orbital cellulitis (*n* = 1) which was successfully treated with antibiotics, and oscillopsia during chewing (*n* = 1) for which eventually a patient-specific 3D-printed PEEK reconstruction was performed. No complications of the other cranial nerves or surgical mortality were observed. After 1-year follow-up two patients developed MRI established growth of residual tumor, for which one patient received photon radiotherapy 1.5 years after surgery and one patient received proton beam therapy 4.0 years after surgery. As stated before, one patient suffered from strong deterioration of visual fields (− 23.1 dB), requiring reresection and proton beam therapy, which improved and stabilized the patient’s visual field deficit (− 10.0 dB). One patient required reresection for the development of ophthalmoplegia, which improved the patient’s symptoms. In these four patients the optic canal was decompressed in one and the orbital roof in three patients.

### Predictors of short- and longer-term postoperative visual acuity, visual fields, and proptosis

#### Short-term

Predictor for worse short-term postoperative BCVA was worse preoperative BCVA: for each point lower preoperative BCVA, postoperative BCVA was 0.49 lower (95%CI − 0.21 to − 0.77, *p* = 0.002). No predictors were identified for short-term visual fields. Predictor of worse postoperative proptosis was worse preoperative proptosis: for each additional millimeter preoperative proptosis, postoperative proptosis was 0.47 mm higher (95%CI 0.16 to 0.78, *p* = 0.006). Detailed information about predictors of short-term outcomes is provided in Supplementary Table 1.

#### Longer-term

Predictors for worse longer-term BCVA were worse preoperative BCVA (*β* = − 0.49, 95%CI − 0.21 to − 0.77, *p* = 0.002), and the number of tumors, as for each extra diagnosed meningioma postoperative BCVA was − 0.14 lower (95%CI − 0.26 to − 0.02, *p* = 0.021). Predictors for worse postoperative visual fields were the maximum diameter of preoperative hyperostosis: for each additional millimeter preoperative hyperostosis, postoperative visual fields were 0.39 dB lower (95%CI − 0.67 to − 0.12, *p* = 0.009), and Simpson grade: for each grade increase in Simpson grade, postoperative visual fields were 3.71 dB lower (95%CI − 6.63 to − 0.78, *p* = 0.017). No predictors were identified for longer-term proptosis. Detailed information about predictors of longer-term outcomes is provided in Supplementary Table 2.

## Discussion

In a recent cohort of spheno-orbital meningioma patients operated by a dedicated team of a neurosurgeon and orbital surgeon in a high-volume referral center good visual outcomes were achieved and maintained with modest morbidity and no mortality. Postoperative visual acuity and visual fields endorsed surgery of patients with SOM, even with minimal visual impairment or hyperostosis, as we showed with our regression analysis that preoperative visual deficits and the maximum diameter of hyperostosis were predictors of poorer outcome.

Results of this mono-center study were in line with published studies of the last two decades, which reported improvement of vision in 37–87% of patients, visual fields in 17–88%, proptosis in 60–100%, and permanent complications in 22–44% of patients [6, 14, 16, 28, 34–36]. We reported improvement of visual acuity in 80% and visual fields in 100% of patients with stable outcomes in 95% of these patients during our modest follow-up period. Proptosis was also improved in all patients; however, 21% reported deterioration at longer follow-up. We observed permanent complications in 32%. Despite the good visual outcomes, 21% of patients showed progression requiring reresection, which was comparable to the outcomes (22–48%) of recently published studies by other groups [6, 9, 25, 36].

### Predictors of postoperative vision

Based on our results, multiple data–driven recommendations can be made to optimize surgery and postsurgical follow-up for SOM patients (Table 3). Our results suggest that it might be beneficial to operate patients, even with minimal visual impairment or hyperostosis, to prevent the development of visual deficits, that might not completely resolve after surgery (i.e., strongest predictors for postoperative visual outcomes were preoperative visual function and hyperostosis), which is in line with conclusions reported in published literature [9, 16, 19, 21, 23, 34, 36, 37]. Our, relatively short, follow-up results suggest early surgery has a lasting change on the clinical course of the disease, with persisting good visual outcomes in the majority of patients. Patients with normal visual function, operated for their proptosis, maintained good visual outcomes after surgery. While surgery of patients with minimal visual symptoms seems intuitive and was recommended by other case series, these studies did not systematically assess predictors of postoperative visual outcomes [9, 16, 19, 21, 23, 34, 36, 37]. As these tumors tend to invade the bone near the foramina of the cranial nerves, early surgery might prevent extensive hyperostosis, narrowing of formina, and consequently cranial nerve deficits [16, 22]. Indeed, it is reported that optic canal and intraorbital involvement are predictors for postoperative visual deficits [37]. Nevertheless, we also acknowledge that surgery itself imposes a risk of new visual and cranial nerve deficits [6, 28]. Especially in very old patients, patients with severe comorbidities, or patients with extensive disease resulting in full blindness, the benefits of surgery might not always outweigh the risk of complications. However, in general, we believe that the risk for new complications might be smaller when patients are operated on early in their disease course, as cranial nerves are less vulnerable when compression is less severe. Our results also indicate that patients diagnosed with multiple intracranial meningioma were at higher risk for postoperative visual acuity deficits. Therefore, we advise a more intensive multidisciplinary postsurgical follow-up for these patients to identify objective or subjective postoperative visual deterioration as early as possible, enabling early reresection. The need for repeat intervention was high in this group.

**Table 3:** Recommendations for surgical indication, surgical technique and patient follow-up

Current practice	Recommendations	Evidence current study	Literature supporting recommendation
Indication for surgery			
▪ Significant visual symptoms or proptosis	▪ Prevention of visual deficits by early surgery, even of patients with minimal visual impairment or hyperostosis	▪ Worse preoperative deficits were related to worse postoperative outcomes	▪ [9, 16, 19, 21, 23, 34, 36, 37]
Surgical technique			
▪ Resection of hyperostotic bone ▪Limited resection of intraorbital meningioma and periorbita ▪ Reconstruction in some patients	▪ Maximum resection of hyperostotic bone: at least the lateral orbital wall, orbital roof, optic canal, and superior orbital fissure ▪ Maximum intraorbital meningioma resection, including periorbita ▪ Reconstruction with titanium mesh or customized 3d-printed PEEK implant	▪ Need for reresection or radiotherapy was observed in patients without decompression of orbital roof and optic canal ▪ Simpson grade was predictive for long-term visual field deficits ▪ Reconstruction with titanium mesh or 3D-printed PEEK implant showed good postoperative proptosis results	▪ [6, 19, 28, 34] ▪ [12, 19, 20, 25, 32, 36] ▪ [2, 6, 8, 14, 19, 34, 36]
Patient follow-up			
▪ Routine meningioma follow-up	▪ More frequent follow-up of patients with multiple meningioma	▪ Tumor number was predictive for long-term visual acuity	▪ No relevant literature

### Surgical approaches

Although multiple surgical approaches have been described for SOM surgery, the pterional approach is the most used approach in these patients, and also used for all patients described in this study [15, 16, 19, 24, 28, 35]. Advantages of pterional craniotomy are wide exposure and access to the anterior, middle, and temporal cranial fossa, and therefore ability to resect the hyperostotic bone and soft-tissue tumor as radically as possible. Recently, multiple endoscopic approaches have been described for anterior skull base pathology, such as the supraorbital, and the combined endonasal and transorbital approach [5, 7, 13, 17, 18, 27, 41]. Three studies described a total of 12 SOM patients operated with the endonasal transorbital approach [1, 5, 18]. The endonsasal approach was used for decompression of the medial part of the optic canal. Further decompression of the hyperostotic bone and tumor removal was accomplished with the transorbital approach [1, 5, 18]. Compared with endonasal approach only, this combined approach enabled resection of more laterally located pathology [5]. Overall these case series showed stabilization of visual function with moderate to good reduction of proptosis. Proposed advantages are the less invasive approach with cosmetically pleasing results. However, gross total resection is often not possible, and therefore, these approaches should be preserved for selected patients with suspected benign meningioma with minimal intradural growth and in whom relief of symptoms through decompression of the optic canal is the primary goal [1]. In these cases residual tumor can be controlled by radiotherapy [1].

### Hyperostotic bone resection, dealing with the periorbita, and reconstruction techniques

In the last decades a paradigm shift has occurred in skull base surgery from aiming maximum surgical resection to optimizing patient outcomes and health-related quality of life [39, 40]. A maximum resection of hyperostotic bone is advocated to reduce proptosis, to restore visual function, and to minimize progression. However, there is no consensus on the degree of bony resection, the need to resect invaded periorbit, and the need for reconstruction of the lateral orbital wall. We agree with earlier reports that cavernous sinus involvement is a contra-indication for gross-total resection [16, 22, 24, 36]. Some of the same reports advise no decompression of superior orbital fissure tumor involvement. However, with transection of the meningo-orbital band, full decompression of the superior orbital fissure is possible [10]. It remains controversial whether resection of bone should be limited to clearly visible hyperostotic bone or whether decompression of the optic canal and possible other foramina should be performed routinely for preservation of good visual function [36]. We recommend resection of at least the orbital roof and lateral orbital wall, and decompression of the optic canal, and superior orbital fissure to prevent further deterioration of visual outcomes and improve proptosis (Table 3). Although standard resection of the anterior clinoid process is performed by others, we only advise to resect this structure in case of hyperostosis to prevent early postoperative progression, as no cranial nerves are directly affected by hyperostosis of the anterior clinoid process [2, 19, 22, 23, 28, 36]. Another debate is the need for resection of the periorbit. While this should clearly be done when the periorbit is invaded with tumor, it is advocated by some to preserve the periorbit to prevent pulsatile enopthalmos. However, we agree with others that resection of the periorbit is critical to maximally reduce proptosis. Based on our own experience and the reported literature, we advise reconstruction with titanium mesh or customized patient-specific 3D PEEK implants to prevent (pulsatile) enopthalmos, especially in case of periorbit resection [2, 14–16, 19, 23, 31, 36]. Other groups have reported to actually not perform reconstruction to provide an even greater reduction of proptosis [22, 28, 35].

### Progression and adjuvant treatment

In this case series with limited follow-up length, 21% of patients needed reintervention. Two patients showed established tumor growth without the development of new visual deficits. These patients were treated with radiotherapy to halt the tumor growth. While radiotherapy is associated with optic neuropathy, extra-ocular muscle dysfunction, and pituitary insufficiency [6, 25], irradiation was chosen over reoperation, as the growing tumor remnants were deemed difficult to fully resect. Especially with the introduction of proton beam therapy, irradiation might be less harmful than reoperation for cases with residual disease or tumor regrowth without symptoms of newly developed visual deficits [4]. However, in the two patients with newly developed visual deficits due to postoperative tumor growth, reoperation was chosen in an attempt to decompress the optic system to improve the visual function of the patient. These percentages and treatment strategies for recurrent disease are in line with other case series [2, 6, 28, 33]. Although our case series did not include any patients with a WHO grade II tumor, other authors advise upfront radiotherapy for these patients [6, 29].

### Strengths and limitations

Strengths of this study are the use of a recent cohort of SOM patients operated by a dedicated set team of a neurosurgeon and orbitoplastic surgeon for assessment of short- and longer-term visual outcomes. Furthermore, we prospectively comprehensively assessed visual outcomes not only reporting visual acuity but also standardized measurement of visual fields. Only few studies have been published reporting results of visual fields, while this is a significant symptom for patients, highly correlated with their health-related quality of life [26]. Another strength is the assessment of predictors for postoperative visual outcomes, enabling formulation of recommendations for SOM surgery and patient follow-up. However, due to the small number of patients no multivariable analysis was performed, and ideally, our results should be validated in a larger (international) dataset to ensure robustness of the results. Although we did not perform a direct comparison between patients with an early vs. late stage disease, we formulated that surgery of patients with minimal visual impairment or hyperostosis might provide better postoperative results, as predictors of worse postoperative visual outcomes were worse preoperative visual acuity and a larger diameter of proptosis. While more intuitive, a direct comparison of early vs. later surgery was not possible due to the small patient sample and might actually not be preferred, as it does not take into account the extent of disease and visual status at diagnosis. Longer follow-up is needed to assess more accurate recurrence rates and the long-term outcomes after reresection and radiotherapy.

## Conclusions

The aim of surgery for spheno-orbital meningioma should be to optimize visual outcomes and health-related quality of life. As spheno-orbital meningioma is a rare disease with significant treatment variation, sound comparison of different treatment strategies and outcomes can only be performed through international collaboration and harmonized data collection. In lack of that, we present outcome data of our recent small series and make an argument for surgical intervention of spheno-orbital meningiomas, even in patients with limited visual impairments or hyperostosis, as worse preoperative visual acuity, and greater diameter of hyperostosis were predictors of poorer visual outcome.

## Electronic supplementary material

ESM 1 (DOCX 14 kb)
