# Supplementary material for: Visual outcomes endorse surgery of patients with spheno-orbital meningioma with minimal visual impairment or hyperostosis
Source: Acta Neurochir (Wien). 2020 Sep 4;163(1):73–82. doi: 10.1007/s00701-020-04554-9 (PMC7778613; doi:10.1007/s00701-020-04554-9)
Supplement: Supplementary file 1 — (DOCX 14 kb) [file 701_2020_4554_MOESM1_ESM.docx]

**Supplementary Table 1: baseline predictors for short-term (3-months) postoperative best corrected visual acuity (BCVA), visual fields and proptosis**

|  | **Beta** | **95%CI** | **p-value** |
| --- | --- | --- | --- |
| **BCVA** |  |  |  |
| Tumor diameter in mm | -0.007 | -0.026 to 0.012 | 0.458 |
| Diameter hyperostosis in mm | -0.005 | -0.026 to 0.016 | 0.630 |
| Simpson grade (I-V) | -0.067 | -0.237 to 0103 | 0.416 |
| Number tumors | -0.120 | -0.245 to 0.005 | 0.059 |
| BCVA (Snellen chart) | 0.487 | 0.207 to 0.766 | 0.002 |
| Age at surgery in years | 0.002 | -0.019 to 0.023 | 0.874 |
|  |  |  |  |
| **Visual Fields** |  |  |  |
| Tumor diameter in mm | 0.041 | -0.074 to 0.156 | 0.441 |
| Diameter hyperostosis in mm | -0.040 | -0.123 to 0.044 | 0.316 |
| Simpson grade (I-V) | -0.020 | -0.885 to 0.844 | 0.959 |
| Number tumors | -0.175 | -0.660 to 0.309 | 0.439 |
| Visual field mean deviation in dB | 0.098 | 0.098 to 0.230 | 0.124 |
| Age at surgery in years | -0.023 | -0.089 to 0.044 | 0.466 |
|  |  |  |  |
| **Proptosis** |  |  |  |
| Tumor diameter in mm | 0.049 | -0.039 to 0.136 | 0.255 |
| Diameter hyperostosis in mm | 0.039 | -0.056 to 0.133 | 0.399 |
| Simpson grade (I-V) | 0.343 | -0.437 to 1.123 | 0.366 |
| Number tumors | -0.124 | -0.764 to 0.516 | 0.687 |
| Proptosis in mm | 0.466 | 0.156 to 0.775 | 0.006 |
| *Age at surgery in years* | -0.083 | -0.170 to -0.004 | 0.059 |

**Supplementary Table 2: baseline predictors for long-term (median 2.4 years) postoperative best corrected visual acuity (BCVA), visual fields and proptosis**

|  | **Beta** | **95%CI** | **p-value** |
| --- | --- | --- | --- |
| **Visual Acuity** |  |  |  |
| Tumor diameter in mm | -0.004 | -0.024 to 0.015 | 0.634 |
| Diameter hyperostosis in mm | 0.004 | -0.017 to 0.025 | 0.716 |
| Simpson grade (I-V) | -0.080 | -0.248 to 0.089 | 0.332 |
| Number tumors | -0.143 | -0.261 to -0.024 | 0.021 |
| BCVA (Snellen chart) | 0.489 | 0.210 to 0.767 | 0.002 |
| Age at surgery in years | -0.005 | -0.026 to 0.015 | 0.596 |
|  |  |  |  |
| **Visual Fields** |  |  |  |
| Tumor diameter in mm | -0.009 | -0.366 to 0.348 | 0.959 |
| Diameter hyperostosis in mm | -0.393 | -0.670 to -0.116 | 0.009 |
| Simpson grade (I-V) | -3.705 | -6.633 to -0.777 | 0.017 |
| Number tumors | 0.508 | -2.231 to 3.247 | 0.695 |
| Visual field mean deviation in dB | 0.331 | -0.313 to 0.975 | 0.284 |
| Age at surgery in years | 0.174 | -0.170 to 0.519 | 0.294 |
|  |  |  |  |
| **Proptosis** |  |  |  |
| Tumor diameter in mm | 0.066 | -0.054 to 0.186 | 0.262 |
| Diameter hyperostosis in mm | 0.035 | -0.097 to 0.166 | 0.557 |
| Simpson grade (I-V) | 0.514 | -0.551 to 1.580 | 0.323 |
| Number tumors | 0.415 | -0.442 to 1.272 | 0.321 |
| Proptosis in mm | 0.364 | -0.140 to 0.867 | 0.146 |
| Age at surgery in years | -0.048 | -0.178 to 0.082 | 0.446 |
